# Early detection of SARS-CoV-2 variants using genomic surveillance: insights from aircraft wastewater and nasal swabs at Kigali International Airport, Rwanda

**DOI:** 10.1016/j.ijregi.2025.100678

**Published:** 2025-07-06

**Authors:** Misbah Gashegu, Raissa Muvunyi, Jean Pierre Musabyimana, Esperance Umumararungu, Laetitia Irankunda, Chantal Mutezemariya, Arlene Uwituze, Nelson Gahima, John Rwabuhihi, Jean Claude Mugisha, Ayman Ahmed, Noel Gahamanyi, Leon Mutesa, Cecilia A. Prator, Elizabeth A. Landis, Casandra W. Philipson, Nicole Bohme Carnegie, Albert Tuyishime, Isabelle Mukagatare, Noella Bigirimana, Claude Mambo Muvunyi

**Affiliations:** 1 Rwanda Biomedical Centre, Kigali, Rwanda; 2 Africa Center of Excellency for Sustainable Cooling and Cold Chain (ACES), Kigali, Rwanda; 3 School of Medicine and Pharmacy, College of Medicine and Health Sciences, University of Rwanda, Kigali, Rwanda; 4 Pan-Africa One Health Institute, Kigali, Rwanda; 5 Ginkgo Bioworks, Boston, MA, USA

**Keywords:** SARS-Cov2, Surveillance, Traveler genomics

## Abstract

**Objectives:**

The growing threat of emerging infectious diseases necessitates proactive genomic surveillance, particularly, in regions with limited resources and low levels of existing reporting. This study highlights the implementation of a comprehensive genomic surveillance program at the Kigali International Airport and explores the utility of a dual-sample strategy leveraging environmental aircraft wastewater and pooled nasal swab sample types for comprehensive detection and characterization of SARS-CoV-2 lineages being imported into Rwanda.

**Methods:**

Using a combined pooled nasal swab and aircraft wastewater sampling approach resulted in complementary insights in terms of geographic coverage, positivity, and variant characterization.

**Results:**

Mutational profiling in source pooled nasal swabs and aircraft wastewater sample data revealed dynamic shifts in mutation prevalence that corresponded with global patterns. Emerging variant JN.1 was detected early in nasal swab data, demonstrating the power of using genomic surveillance as an early warning system.

**Conclusions:**

These results support the feasibility of pathogen surveillance in high-traffic settings and may help drive interest in expanding programs to include pathogens beyond SARS-CoV-2.

## Highlights

• Combined nasal swabs and aircraft wastewater improve SARS-CoV-2 detection.
• Wastewater and clinical surveillance offer complementary insights.
• Early detection of the emerging variant JN.1 shows surveillance effectiveness.
• Flight duration impacts positivity in wastewater and nasal swab sampling.
• This model can expand to other pathogens and busy transit environments.

## Introduction

The rise of infectious diseases continues to pose a significant threat to global health, especially for vulnerable populations in impoverished communities and for health systems unprepared to manage such crises. This became strikingly evident with the emergence of COVID-19, a zoonotic disease caused by SARS-CoV-2, which rapidly spread across the globe, overwhelming health care infrastructures and exposing the vulnerabilities in public health systems worldwide [1].

In response to this global health emergency, nations were compelled to act swiftly to mitigate the impact of the pandemic and safeguard public health. Within the realm of epidemiologic surveillance, a variety of frameworks were developed to enhance the detection and monitoring of SARS-CoV-2. These approaches ranged from rapid antigen tests to the integration of artificial intelligence for expedited self-diagnosis, as well as the widespread application of polymerase chain reaction (PCR) methods, among others [2].

Amid the global response, Rwanda’s scientific community quickly adopted an innovative approach to SARS-CoV-2 detection, allowing mass testing in low-prevalence settings [3]. This model uses a pooling system and involves merging multiple swab samples into a single sample tube for initial testing. If the pooled sample tests positive, individual tests are subsequently conducted on each sample to identify the infected individuals. This approach optimizes testing resources while enhancing detection efficiency, contributing to Rwanda’s effective pandemic response [3]. Rwanda, in partnership with Ginkgo Biosecurity, has further refined this pooled testing approach by focusing on travel entry points, using passenger nasal swabs and wastewater samples from aircraft to monitor the spread of SARS-CoV-2 variants.

The movement of people through international air travel is a key factor in the global transmission of infectious diseases. In 2019, 4.46 billion passengers traveled by air, a number that underscores the significance of airports as critical sites for monitoring the spread of pathogens [4]. The COVID-19 pandemic highlighted how swiftly a novel pathogen, such as SARS-CoV-2, can be disseminated via air travel, as well as the essential role airports play in the early detection and management of emerging infectious diseases [5,6]. Genomic surveillance efforts, including wastewater sampling from aircrafts, provide a cost-effective and valuable method for monitoring viral diversity and circulation among international travelers [[7], [8], [9], [10]].

Studies have demonstrated that such surveillance can detect SARS-CoV-2 variants in aircraft wastewater 18-31 days before local clinical cases, offering critical early warning data that can inform public health responses [8]. Complementary to wastewater monitoring, voluntary nasal swab testing of arriving travelers further enriches the surveillance system by providing high-resolution genomic data and insights, enabling the detection of variants circulating in populations that may not be captured through wastewater alone [11].

The COVID-19 pandemic also revealed the importance of genomic surveillance in tracking the spread and evolution of the virus. The first confirmed case of COVID-19 in Africa was reported in Egypt in mid-February 2020, and by the end of March, many other countries across the continent had confirmed cases [12]. Epidemiologic analyses demonstrated that the majority of these early cases were linked to air travel, predominantly from Europe [12,13]. Genomic surveillance was a critical component of Africa’s response to these early introductions, and efforts continued to expand over the course of the pandemic. However, significant gaps remain in the spatial and temporal representation of genomic data, particularly, from incoming travelers [14], with sequencing capacity concentrated in only a few African countries.

Rwanda’s early and comprehensive approach to COVID-19, testing, contact tracing, and isolation has been widely recognized. Here, we present the implementation of Africa’s first combined aircraft wastewater and pooled nasal swab pathogen surveillance program, an innovative model that enhances regional preparedness by integrating proven surveillance methods to proactively monitor emerging pathogens.

## Methods

### Nasal swab sample collection

**Figure 1:**
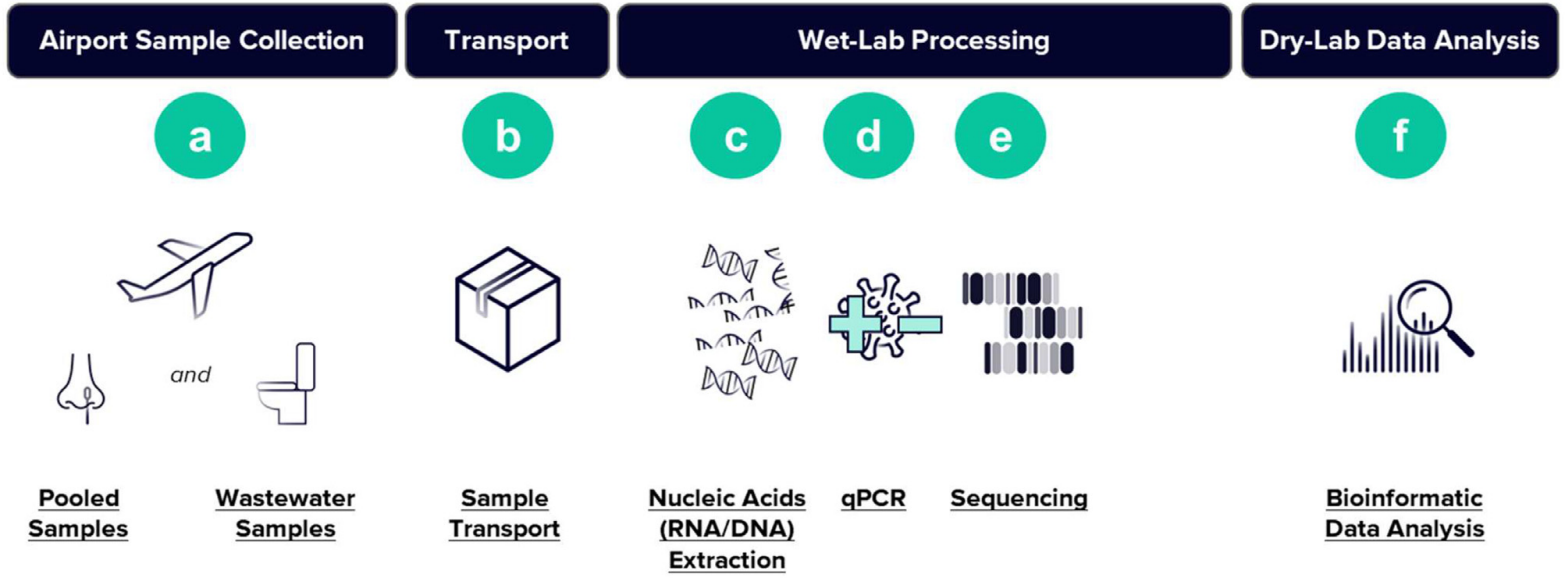
Overview of sample processing workflow. For pooled samples (a), travelers volunteer to collect two nasal swabs at the airport; swabs are grouped in pools (5-10 swabs/pool) and sent to the laboratory. Wastewater samples are collected by aircraft ground handlers from the aircraft lavatories (a). Samples are then transported (b) in temperature-stabilized boxes and shipped overnight to a central laboratory. Once samples have reached the laboratory, nucleic acids are extracted (c). All samples are then tested for SARS-CoV-2 using reverse transcription–qPCR (d). Samples positive for PCR then undergo tiled amplicon sequencing (e). Sequencing data are then analyzed to determine the presence of viral variants (f). qPCR, quantitative polymerase chain reaction.

Nasal swab samples were collected using standardized swab testing kits and a self-swabbing procedure. Participants exiting international flights were recruited on a voluntary basis and provided written informed consent before sample collection. Two swab samples were collected for each participant. For collection, participants were instructed to handle the swabs without touching the sterile tip and to insert the swab into one nostril until the cotton or foam tip was no longer visible, followed by rotation in a circular motion along the entire inner surface of each nostril at least three times before removal. This process was repeated with the second swab. One swab from each participant was placed in a pooled collection tube containing 5-10 swabs, which was labeled with the month, day, flight number, group identifier, and date of collection. The second swab was placed in an individual tube labeled with the flight number and participant confirmation number. A summary of the sample collection workflow is summarized in Figure 1.

### Aircraft wastewater sample collection and transportation

Aircraft wastewater samples were collected using a proprietary wastewater collection device developed by Ginkgo Biosecurity. The device was attached to the lavatory drain hose of the aircraft, and the standard lavatory dumping procedure, as outlined by airline regulations, was initiated. The sample was collected into a sterile sample bottle through a valve on the collection device. This process was repeated for each lavatory tank on the aircraft. Once all samples were collected, the collection bottle was sealed, and the device was flushed. The flushing process involved completing the standard lavatory tank flush procedure in accordance with airline protocols, attaching the glycol hose to the device’s glycol nozzle, and flushing the system with a glycol solution through the valve. Samples were transported to the central laboratory within the day of collection between 2°C and 8°C.

### Extraction, detection by quantitative reverse transcription–polymerase chain reaction, and sequencing

Aircraft wastewater samples were concentrated using Nanotrap microbiome. Particles (Ceres Nanosciences, Inc.) were taken from 1.2 ml of the sample and extracted using the Magmax Microbiome Ultra Nucleic Acid Kit (Thermo Fisher Scientific) on the semi-automated Kingfisher Flex Purification System with a 96 Head version 1.01.0 (Thermo Fisher Scientific, USA). Pooled nasal swab samples (n = 5-10 per pool) were extracted using Maxwell RSC Viral Total Nucleic Acid Purification Kits (Promega) on the benchtop automated Maxwell RSC 48 DNA/RNA extraction platform (Promega, USA). Wastewater and nasal swab samples were eluted in 100 µl of elution buffer according to the protocol.

For nasal swabs and wastewater extracts, samples underwent quantitative reverse transcription-PCR (RT-qPCR) to determine positivity and select samples for downstream sequencing. Nasal samples were tested using the ThermoFisher Taqpath COVID-19 Combo Kit (A47814) according to the manufacturer’s instructions [15]. Nasal samples were considered positive when cycle threshold (Ct) values of the N1 and ORF genes were <37. For wastewater, samples were tested using the GoTaq Enviro Wastewater SCV2 kit (AM2100) and considered positive when Ct values of the N1 gene were <40.

Tiled amplicon sequencing was performed using ARTIC 5.3.2 primer schemes [16] on a Minion sequencer (Oxford Nanopore Technologies) using R9.4.1 flow cells for samples that showed positivity on PCR with the Ct value of 32 or below. Multiplexed sequencing libraries were prepared using a modified version of the NEBNext ARTIC Standard Protocol with PCR Bead Cleanup described in NEBNext ARTIC SARS-CoV-2 Companion Kit (NEB #E7660S/L) Instruction Manual. Sequencing was conducted following the Oxford Nanopore Protocol SQK-LSK109 using high-accuracy base calling models with a 48-hour run duration.

### Bioinformatic analyses

The raw sequencing data were processed through a custom bioinformatics pipeline built for nanopore sequencing. The quality of nanopore sequencing data was assessed using nanoplot 1.40.0, followed by aggregation of pre-demultiplexed reads from MinKNOW/Guppy with ARTIC Guppyplex v1.2.2 [17]. Quality-filtered reads with Q ≥9 were then aligned to the reference genome (MN908947.3) using ARTIC Minion v1.2.2. Unmapped reads were removed, and alignment metrics were obtained with SAMtools v1.15.1.^18^ Downstream variant analysis was done using BCFTools v1.15.1 [18], and variants were annotated using SnpEff v5.0 [19] and SnpSift v4.3 [20]. A consensus genome was generated based on stringent quality criteria, and lineage deconvolution for wastewater samples was conducted using Freyja v1.5.0 [21], with additional lineage identification via Pangolin v4.3.1 [22].

### Phylogenetic analysis

Publicly available sequence data was accessed on September 2, 2024 for SARS-CoV-2 JN.1 sequences from Global Initiative on Sharing All Influenza Data (GISAID) collected between September 26, 2023 and October 24, 2023 [23] (Supplementary Table 1). The JN.1 consensus genome sequence and publicly available GISAID data were aligned with Muscle v3.8.318 [24] and manually verified to ensure data quality. A maximum likelihood phylogenetic tree was generated using the aligned sequences (iqTREE v1.6.12) [25]. Bootstrap analysis was conducted with 1000 replicates to assess the robustness of the phylogenetic tree. The maximum likelihood tree was visualized and annotated using FigTree v1.4.4 [26].

### Statistical analysis

Although flight information was not collected from passengers providing nasal swabs, we can identify the arrival flight for a subset of nasal pools based on the time of collection and the timing of arrivals to Kigali International Airport. For this analysis, flights are only included if they have a wastewater sample and at least one nasal pool that can be attributed to that flight. The majority of such flights (84.5%) had only one associated nasal swab pool and another 13.4% had two associated pools. No flight has more than five associated nasal pools.

The correspondence between nasal swab and wastewater testing results was assessed via correlation. First, indicators for PCR positivity were constructed for each sampling method. In the case of wastewater, this is simply the PCR test result for the single aircraft wastewater sample collected from the flight. For nasal pools, the indicator was positive if at least one pool of samples from passengers on the flight tested positive for SARS-CoV-2. The Pearson correlation between the two positivity indicators was calculated and Pearson chi-square test was used to assess the statistical significance of the correlation. When evaluating the relationship between SARS-CoV-2 PCR positivity and the duration of a flight, each nasal pool was included as a separate data point for this analysis. Covariate-adjusted correlations were obtained via logistic regression.

## Results

### Aircraft wastewater and pooled nasal swab sample collection overview

**Figure 2:**
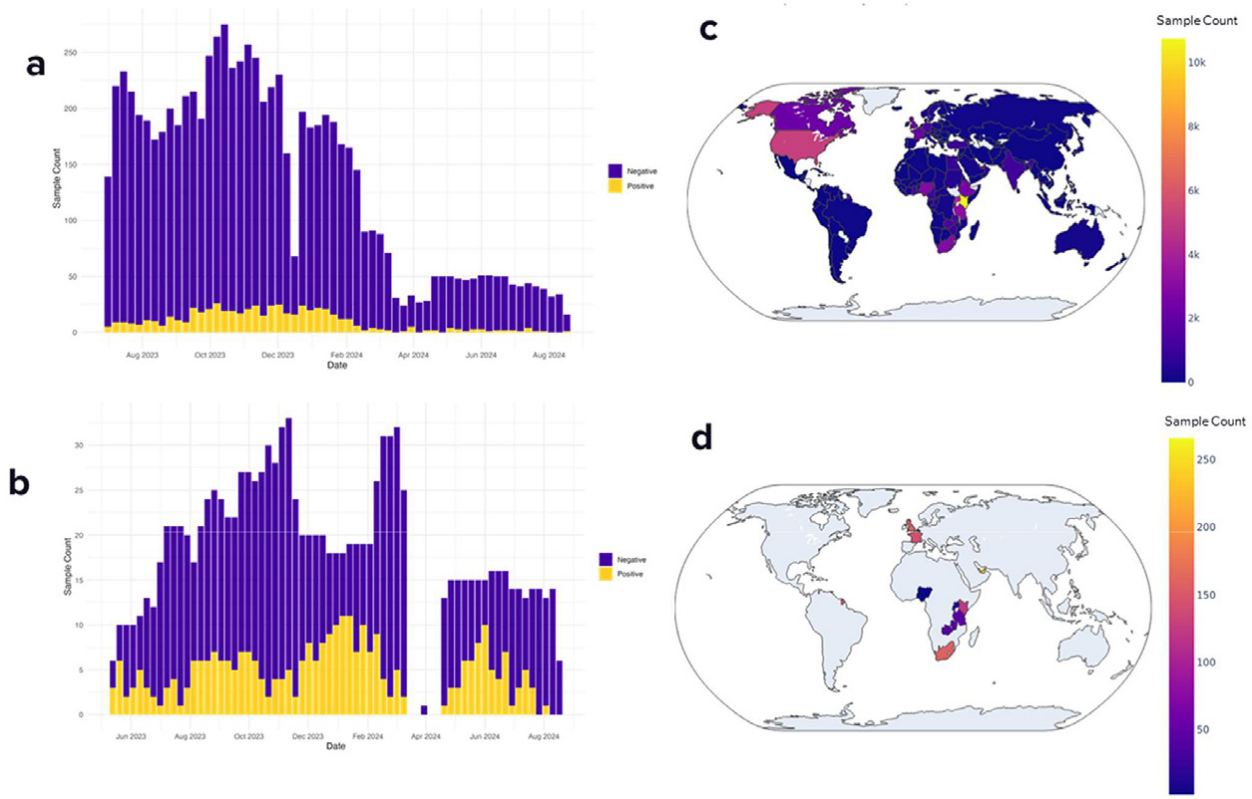
Summary of polymerase chain reaction positivity and sample collection for pooled nasal swabs (a and c) and wastewater (b and d). Bar charts (left) show the number of samples collected by week and whether the sample was positive or negative for SARS-CoV-2 by quantitative reverse transcription–polymerase chain reaction. The maps (right) summarize sample collection breadth of geographic coverage based on flight origin country.

From July 2023 to August 2024, 7869 pooled nasal swab samples with an average pool size of 8.7 individual swabs were collected and tested for SARS-CoV-2 (Figure 2a) using RT-qPCR. Of those, 565 samples tested positive for SARS-CoV-2, and 224 of the positive samples were sequenced. Pooled nasal swab sample collection had a large geographic breadth of coverage, with 169 countries of flight origin represented (Figure 2b). Aircraft wastewater samples were collected from May 2023 to August 2024. Of 1165 aircraft wastewater samples collected, 289 samples tested positive for SARS-CoV-2 using RT-qPCR, and 209 were sequenced (Figure 2c). Aircraft wastewater samples represented flights originating from eight countries (Figure 2d).

### Flight duration impacts SARS-CoV-2 detection across sampling modalities

**Figure 3:**
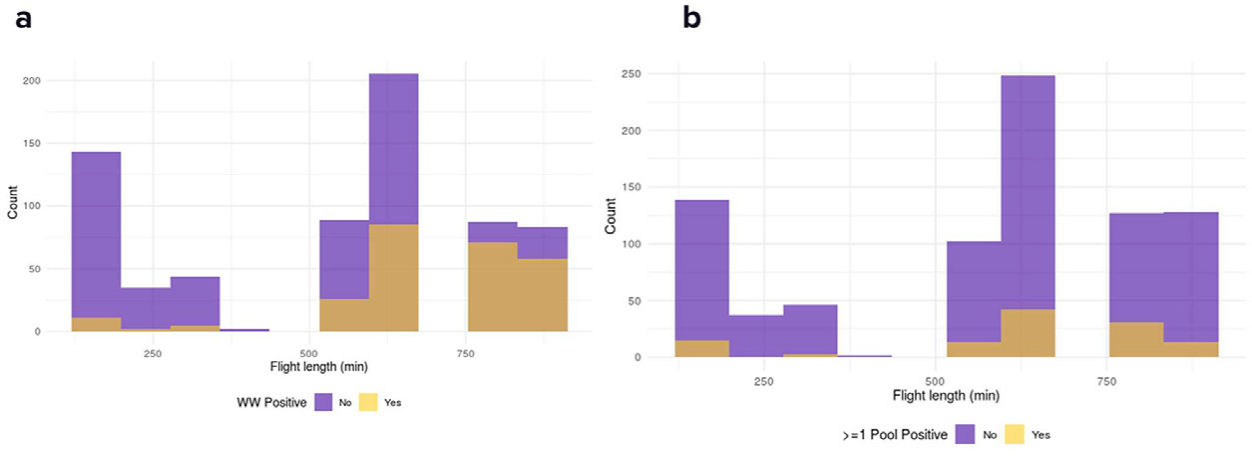
Relationship between flight duration and SARS-CoV-2 detection in wastewater (a) and pooled nasal swabs (b) at Kigali International Airport. (a) In aircraft wastewater, a near-perfect break point was observed at 6 hours (360 minutes), with the percentage of positive samples increased with longer flight durations. (b) For pooled nasal swab samples (b), the number of positive samples increased as flight duration extended. Overall, the detection rate in nasal swabs was lower than aircraft wastewater samples.

The relationship between flight duration and SARS-CoV-2 detection was analyzed in aircraft wastewater (Figure 3a) and pooled nasal swabs (Figure 3b). In wastewater samples, a clear break point was observed at 6 hours (360 minutes), with the positivity rate increasing from 11% overall among shorter flights to 34% among longer flights. The positivity rate in pooled nasal swab samples was consistently lower than in wastewater samples, but positivity increased from 7.5% among flights under 6 hours to 14% among longer flights. Flights longer than 6 hours were associated with significantly higher detection rates in wastewater (odds ratio [OR] = 4.6, *P* <0.0001) and nasal swab samples (OR = 3.1, *P* <0.0001), with wastewater having a higher detection rate overall.

### SARS-CoV-2 detection is independent across sampling modalities

There was little evidence of a marginal correlation between the two sampling methods (chi-square test of independence, *P* = 0.14; OR = 1.46, 95% confidence interval: 0.87-2.44). As seen previously, sampling methods showed a significant association with flight duration, suggesting that what little correlation is observed may be due to confounding by flight length. After adjustment for flight duration, the evidence of a correlation between wastewater and nasal swab results was even weaker (*P* = 0.72; adjusted OR = 1.08).

These findings indicate that although longer flight times increase the likelihood of detecting SARS-CoV-2 for both sampling methods, wastewater and nasal swab positivity rates are largely independent of each other by flight and that the combined use of these sampling methods increases the overall likelihood of detecting SARS-CoV-2.

### Diversity of SARS-CoV-2 variants captured in aircraft wastewater and nasal swab sequencing

**Figure 4:**
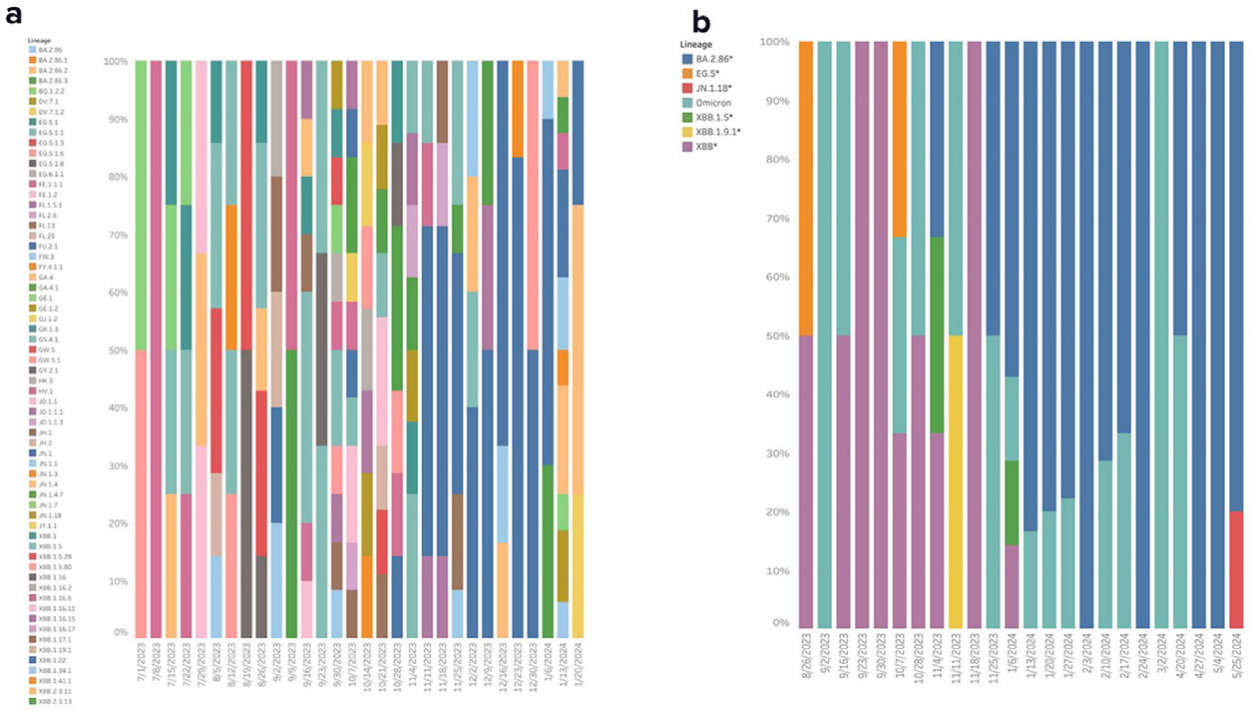
Diversity and abundance of SARS-CoV-2 variants detected by collection date through pooled nasal swab (a) and aircraft wastewater (b) sequencing from July 2023 to May 2024.

Aircraft wastewater and nasal swab samples selected for sequencing were used to monitor for emerging SARS-CoV-2 variants and mutations. SARS-CoV-2 lineages were characterized if reference genome coverage was ≥70%. A total of eight major variants (XBB.1.5, XBB.2.3, XBB.1.9.1, JN.1, XBB.1.9.2, XBB.1.16, EG.5, and BA.2.86) were detected in the nasal swab samples collected (Figure 4a) and six major variants (XBB, XBB.1.5, XBB.1.9.1, EG.5, BA.2.86, and JN.1) were identified in aircraft wastewater (Figure 4b) from July 2023 to August 2024. Of the total samples sequenced, lineages could be identified in 84% of pooled nasal swabs (n = 188 of 224) and 64% of aircraft wastewater samples (n = 133 of 209).

**Figure 5:**
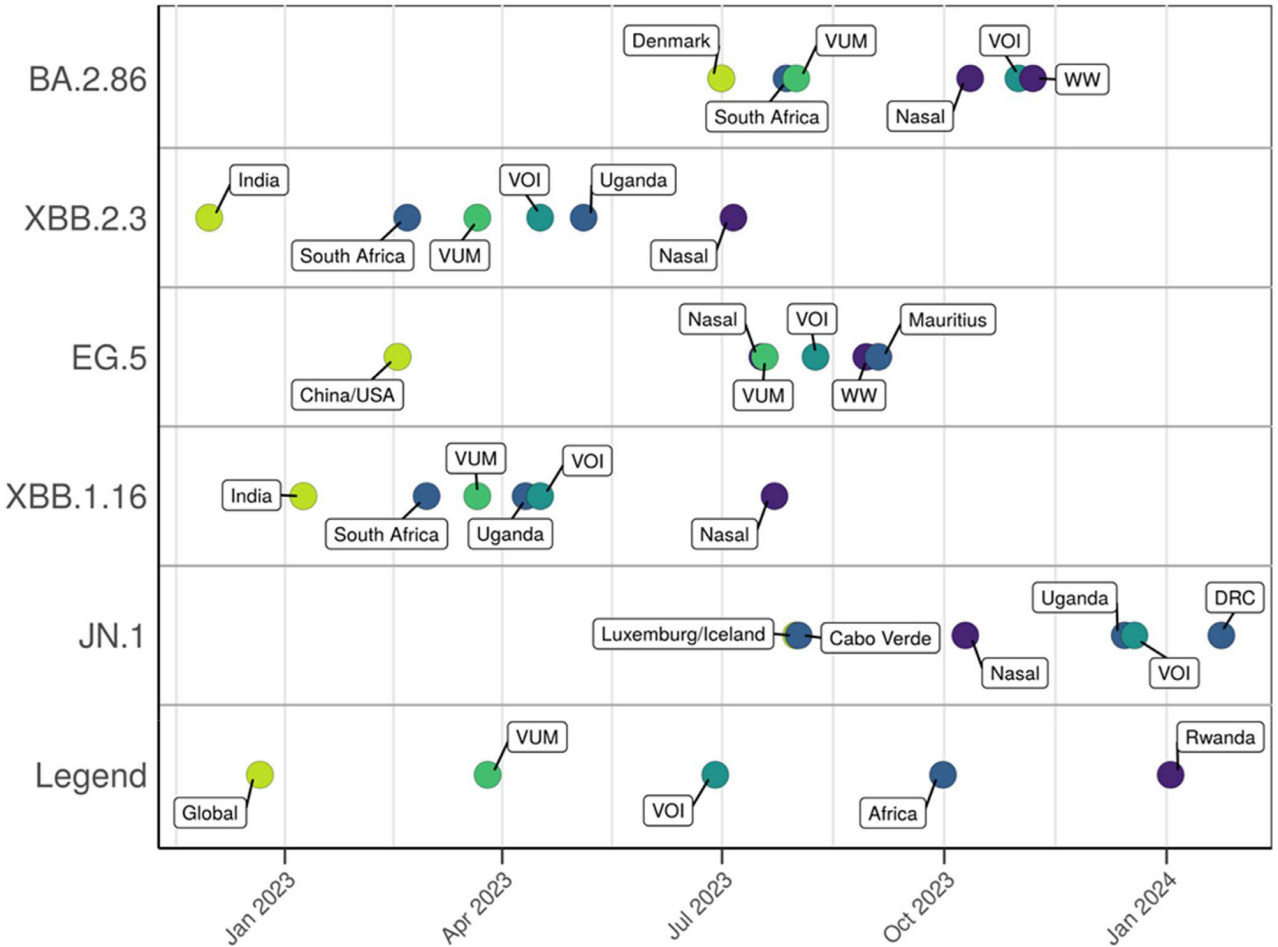
Timeline of collection dates of samples sequenced by this program alongside their first detections worldwide, in Africa, and in countries neighboring Rwanda. DRC = Democratic Republic of Congo; Global = first location a respective variant was detected worldwide; VOI = World Health Organization designated variant of interest; VUM = World Health Organization–designated variant under monitoring; WW = wastewater sample.

Several of the variants of SARS-CoV-2 detected were designated by the World Health Organization (WHO) as variants of interest (VOIs) or variants under monitoring (Figure 5). The timeline in Figure 5 illustrates the collection dates of samples sequenced by this program alongside their first detections worldwide, in Africa, and in countries neighboring Rwanda. Due to the timing of these surveillance efforts, which began mid-pandemic, JN.1 and BA.2.86 were the only major variant introductions captured in real time. JN.1 was detected in a pooled nasal swab sample collected on October 10, 2023. Using phylogenetics, its sequence was compared with all JN.1 genomes deposited into GISAID within the 2 weeks of the variant’s detection (September 26, 2023 to September 24, 2023) (Figure S1). For the entire phylogenetic tree, see Supplementary Figure 1. The phylogenetic tree shows the Rwanda JN.1 cluster with a sample collected in Ireland on October 19, 2023, suggesting that this lineage may have been introduced to Rwanda by a traveler from Europe. The clade comprising the sequence from Ireland and the Rwanda sequence received strong bootstrap support (87%), indicating a statistically well-supported evolutionary relationship. This robust clustering suggests that these two sequences are likely closely related. Considering the broader context of all available sequence data from public databases, the results further imply that the Rwanda JN.1 strain is evolutionarily and geographically closely linked with the Ireland strain.

Mutational profiling was used to track nine key defining mutations for XBB and BA.2.86, enabling detailed tracking of these variants’ evolution. BA.2.86-defining mutations (S:R356T, S:L455S, and S:E554K) were present in source pooled nasal swabs and aircraft wastewater sample data and increased in frequency with the onset of the BA.2.86 global wave (Figure S2). JN.1-defining mutation S:L455S appeared in October 2023 in nasal swab data corresponding with the timing of this program’s first JN.1 detection. XBB-defining mutations (S:R346T, S:F456L, and S:L455F) were also present in both sampling modalities and decreased as BA.2.86 and JN.1 increased in prevalence over the sample collection period.

## Discussion

This study describes the outcomes of the first-of-its-kind aircraft wastewater surveillance program in Africa and highlights SARS-CoV-2 detection and characterization in aircraft wastewater and pooled nasal swab samples collected between July 2023 and August 2024. Our findings emphasize the utility of two distinct sampling modalities for monitoring SARS-CoV-2 on a global scale and highlight their complementary nature in capturing SARS-CoV-2 variants arriving at Rwanda’s Kigali International Airport.

Overall, pooled nasal swab sampling demonstrated a broad geographic reach, with samples representing passengers reporting 169 different countries of origin. In contrast, aircraft wastewater was sampled from flights originating in eight countries, which reflects the challenge that wastewater samples cannot always be associated with the countries of origin of all passengers. Using historical bookings data, we estimate that the sampled flights included passengers from 103 different countries of origin. Our analysis revealed a strong association between flight duration and the likelihood of SARS-CoV-2 detection for both sampling methods. Flights longer than 6 hours were associated with a nearly five-fold increase in the odds of detection in wastewater and a three-fold increase in the odds of detection in nasal swabs. It is worth noting that short- and long-haul flights necessarily originate in different sets of countries, so differences in SARS-CoV-2 burden in the populations captured on short vs long flights could explain some of this difference. In addition, nasal pools corresponding to long-haul flights tended to include more samples, which would necessarily increase the odds of detection. All together, these findings suggest that, at least in wastewater, longer flights provide greater opportunities for viral shedding and that flight duration is a significant factor in designing surveillance strategies. A previous study found similar results confirming that an individual’s likelihood to defecate was higher on long-haul flights greater than 6 hours in duration (a shift from <13% vs <36% of the total passengers in short- vs long-haul flights, respectively), supporting the connection between flight duration and viral shedding [27].

Comparing sampling modalities, minimal correlation was observed between wastewater and nasal swab testing (*P* = 0.14), particularly, after adjusting for flight length (*P* = 0.72). This suggests that the two testing methods are largely independent. Although we would expect the testing results to be strongly correlated if both tests were performed on the same samples or the same set of passengers, here, testing is on two unrelated samples from different subsets of passengers on a given flight. Thus, pooled nasal swab and aircraft wastewater sampling serve as complementary approaches wherein SARS-CoV-2–positives that may be missed by one method can be detected by the other. We expect the detection rate in wastewater to be higher than in nasal swabs because a nasal swab pool in this program captures at most 10 travelers, whereas aircraft wastewater can theoretically capture all passengers on a flight. Given this, wastewater testing may be particularly valuable for capturing infections on longer flights where viral shedding into wastewater is more pronounced.

The sequencing results further underscore the value of integrating aircraft wastewater and nasal swab testing strategies to track a diverse array of SARS-CoV-2 variants. Eight major variants, including WHO-designated VOI XBB.1.5, EG.5, JN.1, and BA.2.86, were detected in pooled nasal swab samples, whereas six variants were identified in aircraft wastewater. Importantly, variant detection rates were relatively high, with lineages identified in 84% of nasal swab samples and 66% of wastewater samples. In the case of JN.1, this lineage was detected in a pooled nasal swab collected on October 10, 2023, months before it was designated as a VOI by the WHO on December 19, 2023. In addition, mutational profiling identified JN.1 receptor-binding domain mutation (L455S) could also be detected as early as October, suggesting that tracking such mutations as warning signals of emerging SARS-CoV-2 variants. Together, these findings provide a framework for monitoring viral transmission and variant emergence in the context of global travel, enhancing our ability to detect threats early.

The successful implementation of this comprehensive surveillance program marks a significant advancement in Rwanda’s public health infrastructure, particularly, in its ability to monitor and respond to infectious diseases introduced through international travel. Expanding genomic surveillance to other high-traffic international airports and settings such as land borders could enhance global preparedness and response to future pandemics, particularly, in regions with limited genomic surveillance resources. Building on this success, the Rwanda Biomedical Center plans to expand its airport surveillance to include an extensive panel of pathogens in addition to SARS-CoV-2.

## Declarations of competing interest

CAP, EAL, CWP, and NBC are employed by Ginkgo Bioworks and own Ginkgo Bioworks employee stocks and/or Restricted Stock Units grants.

## Appendix Supplementary materials

Image, application 1

Image, application 2

Image, application 3

Image, application 4

Image, application 5

Image, application 6

## Footnotes

Supplementary material associated with this article can be found, in the online version, at doi:10.1016/j.ijregi.2025.100678.
