# Supplementary figures and images for: Early detection of SARS-CoV-2 variants using genomic surveillance: insights from aircraft wastewater and nasal swabs at Kigali International Airport, Rwanda
Source: IJID Reg. 2025 Jul 6;16:100678. doi: 10.1016/j.ijregi.2025.100678 (PMC12269423; doi:10.1016/j.ijregi.2025.100678)

## Slide 1
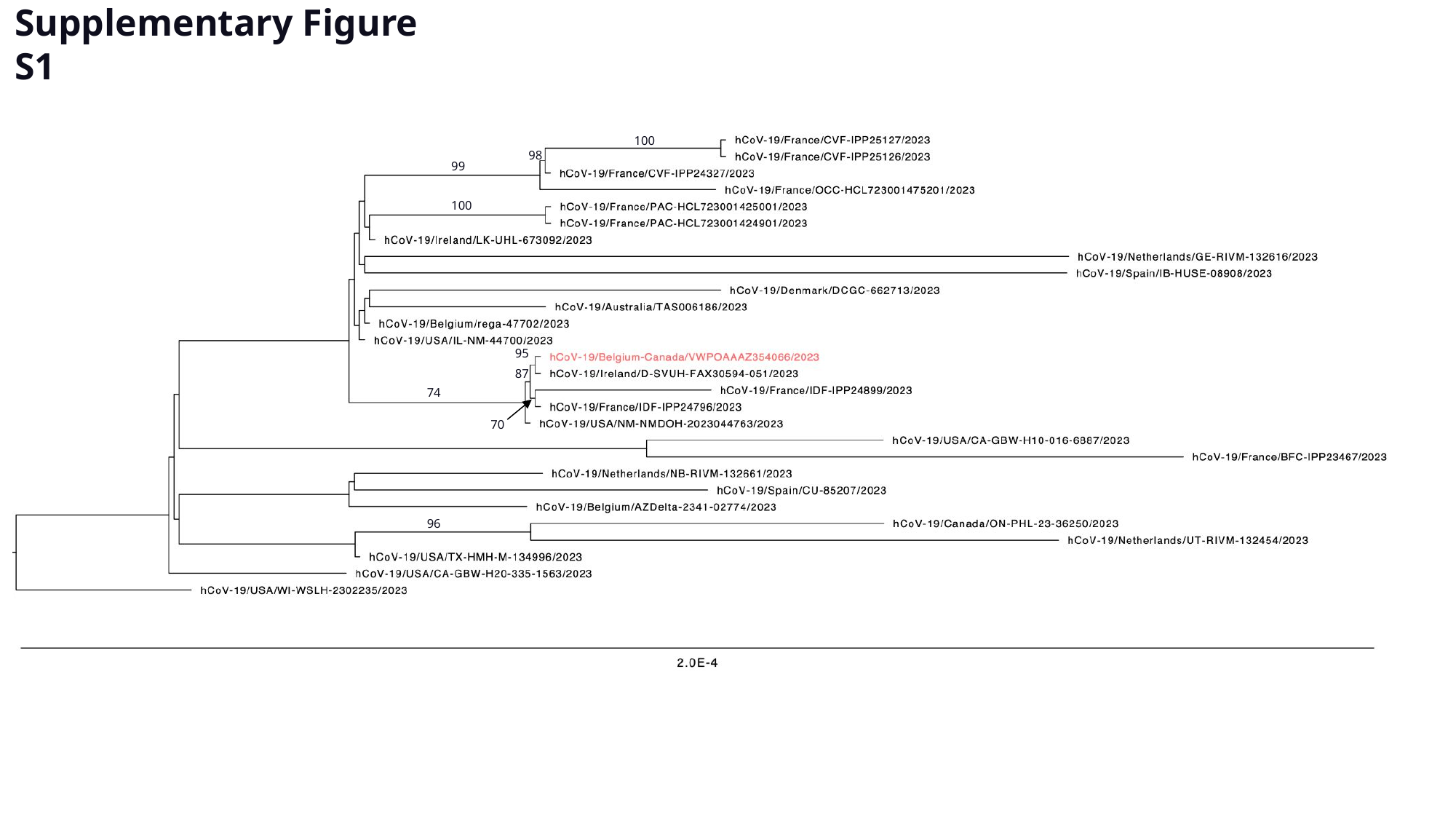

Supplementary Figure S1
100
98
99
100
95
87
74
70
96

## Slide 2
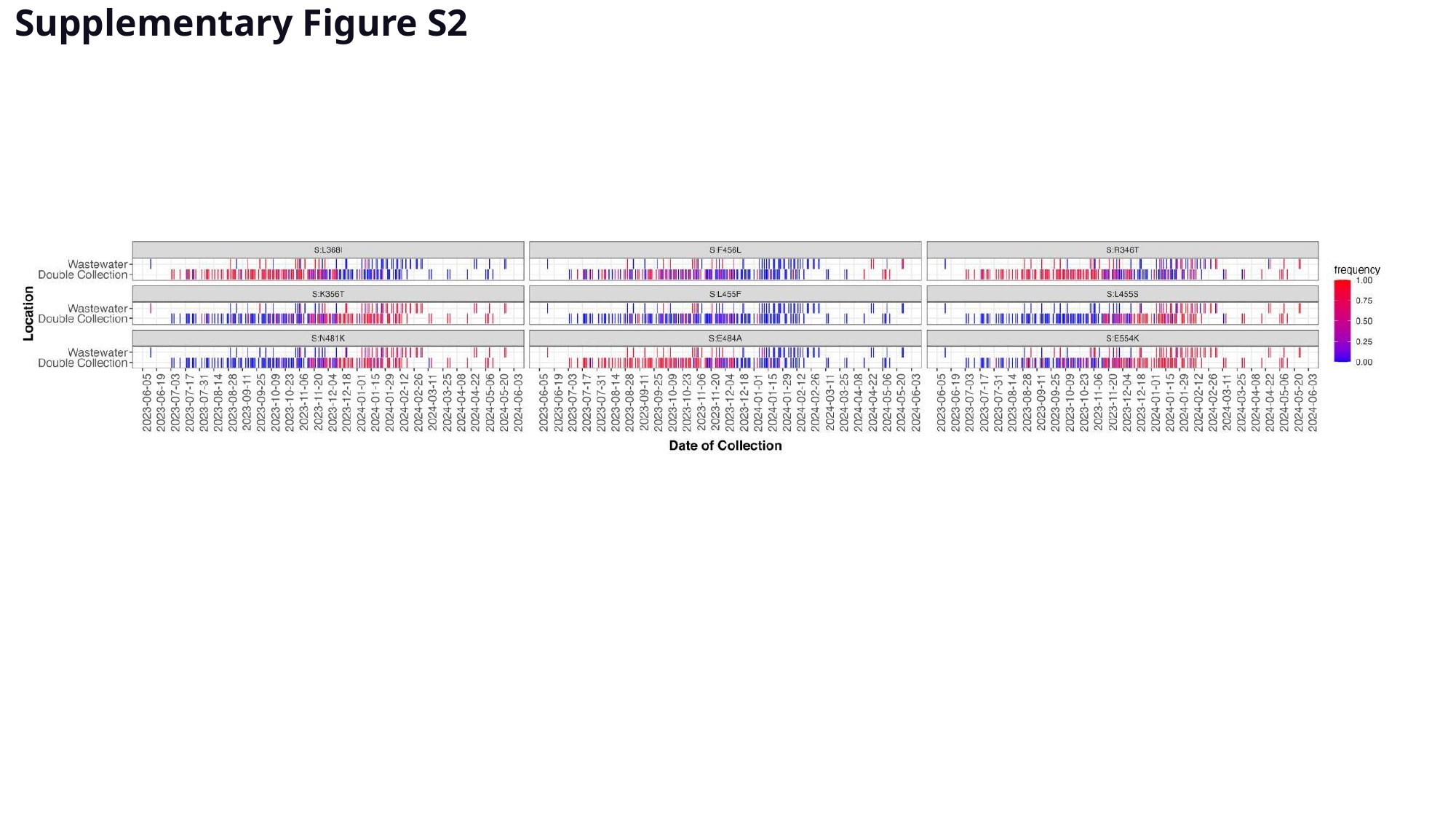

Supplementary Figure S2

Supplement: Supplementary file 2 [file mmc2.pptx]
