# Supplementary material for: Early detection of SARS-CoV-2 variants using genomic surveillance: insights from aircraft wastewater and nasal swabs at Kigali International Airport, Rwanda
Source: IJID Reg. 2025 Jul 6;16:100678. doi: 10.1016/j.ijregi.2025.100678 (PMC12269423; doi:10.1016/j.ijregi.2025.100678)
Supplement: Supplementary file 3 [file mmc3.docx]

**Supplementary Figure S1. Phylogenetic tree of first JN.1 detected in Rwanda Biomedical Center’s genomic surveillance program with representative global data with collection dates within two weeks.** The clade that includes the sequence from Ireland and the sequence from Rwanda (red) received strong bootstrap support (87%), highlighting a statistically well-supported evolutionary relationship.

**Supplementry Figure S2. Frequency of select mutations defining for XBB and BA.2.86 in aircraft wastewater and pooled nasal swabs.** Heat map of mean frequency by collection date for each mutation and sampling modality.
