# Supplementary material for: Early detection of SARS-CoV-2 variants using genomic surveillance: insights from aircraft wastewater and nasal swabs at Kigali International Airport, Rwanda
Source: IJID Reg. 2025 Jul 6;16:100678. doi: 10.1016/j.ijregi.2025.100678 (PMC12269423; doi:10.1016/j.ijregi.2025.100678)
Supplement: Supplementary file 4 [file mmc4.docx]

**Supplementary Table 1.** Metadata of publicly available SARS-CoV-2 JN.1 sequences accessed from GISAID on 2024-09-02, comprising samples collected between 2023-09-26 and 2023-10-24. Columns include strain name, virus, GISAID accession ID, collection date, geographic origin (region, country, division, location), exposure information, genomic segment, sequence length, host details (age and sex), and associated laboratory and submission metadata (originating lab, submitting lab, authors, and date submitted).
